# Neonatal Gram Negative and Candida Sepsis Survival and Neurodevelopmental Outcome at the Corrected Age of 24 Months

**DOI:** 10.1371/journal.pone.0059214

**Published:** 2013-03-18

**Authors:** Timo R. de Haan, Loes Beckers, Rogier C. J. de Jonge, Lodewijk Spanjaard, Letty van Toledo, Dasja Pajkrt, Aleid G. van Wassenaer-Leemhuis, Johanna H. van der Lee

**Affiliations:** 1 Department of Neonatology, Emma Children’s Hospital, Academic Medical Center Amsterdam, The Netherlands; 2 Department of Neonatology, Erasmus MC – Sophia Children’s Hospital, Rotterdam, The Netherlands; 3 Department of Microbiology, Academic Medical Center, Amsterdam, The Netherlands; 4 Department of Pediatric Infectious Diseases, Academic Medical Center Amsterdam, The Netherlands; 5 Department of Pediatric Clinical Epidemiology, Emma Children’s Hospital, Academic Medical Center, Amsterdam, The Netherlands; NIAID, United States of America

## Abstract

**Objectives:**

To evaluate the long term neurodevelopmental outcome of premature infants exposed to either gram- negative sepsis (GNS) or neonatal Candida sepsis (NCS), and to compare their outcome with premature infants without sepsis.

**Methods:**

Historical cohort study in a population of infants born at <30 weeks gestation and admitted to the Neonatal Intensive Care Unit (NICU) of the Academic Medical Center in Amsterdam during the period 1997–2007. Outcome of infants exposed to GNS or NCS and 120 randomly chosen uncomplicated controls (UC) from the same NICU were compared. Clinical data during hospitalization and neurodevelopmental outcome data (clinical neurological status; Bayley –test results and vision/hearing test results) at the corrected age of 24 months were collected. An association model with sepsis as the central determinant of either good or adverse outcome (death or severe developmental delay) was made, corrected for confounders using multiple logistic regression analysis.

**Results:**

Of 1362 patients, 55 suffered from GNS and 29 suffered from NCS; cumulative incidence 4.2% and 2.2%, respectively. During the follow-up period the mortality rate was 34% for both GNS and NCS and 5% for UC. The adjusted Odds Ratio (OR) [95% CI] for adverse outcome in the GNS group compared to the NCS group was 1.4 [0.4–4.9]. The adjusted ORs [95% CI] for adverse outcome in the GNS and NCS groups compared to the UC group were 4.8 [1.5–15.9] and 3.2 [0.7–14.7], respectively.

**Conclusions:**

We found no statistically significant difference in outcome at the corrected age of 24 months between neonatal GNS and NCS cases. Suffering from either gram –negative or Candida sepsis increased the odds for adverse outcome compared with an uncomplicated neonatal period.

## Introduction

Nosocomial infections caused by gram negative and Candida sepsis in the Neonatal Intensive Care Unit (NICU) may lead to overt clinical sepsis increasing the risk for mortality, co- morbidity and long-term neurodevelopmental sequelae [1]–[3]. Extremely Low Birth Weight (ELBW) and Very Low Birth Weight (VLBW) infants are the most fragile and immunologically compromised patients in the NICU [2], [4], [5]. Well known risk factors for nosocomial sepsis in this population are surgery (mostly for Necrotising Entero Colitis, NEC), prolonged ventilatory support, the use of total parenteral nutrition (TPN), indwelling central venous catheters (CVC), corticosteroid medication use and the frequent use of broad-spectrum antibiotics [2], [6]–[10].

Sepsis episodes caused by gram- negative bacteria (gram- negative sepsis; GNS) and neonatal Candida sepsis (NCS) are both associated with high mortality and morbidity rates, ranging from 19–36% and 32–44% respectively [1], [2], [9]–[11]. The reported cumulative incidence of GNS in the NICU varies from 2.7 to 4.4% [3], [12], [13], compared with 1.7 to 9.0% [3], [6], [10], [12] for NCS. In GNS *Escherichia coli* and *Klebsiella pneumoniae* are the most frequently encountered microorganisms [7], [14], [15]. Most commonly reported causative microorganisms in NCS are *Candida albicans* and *Candida parapsilosis* [6], [16].

Long- term neurological sequelae of GNS and NCS in VLBW infants have been reported in 45% and 57% of exposed infants respectively [3]. The presence of meningitis during neonatal sepsis is a major additional risk factor for adverse outcome [4], [17].

Reports on the long-term outcome of infants suffering nosocomial sepsis are sparse. The aim of this study was therefore to investigate the odds for adverse outcome (death or severe neurodevelopmental delay) in infants with a gestational age <30 weeks admitted at a tertiary NICU suffering either GNS or NCS in comparison with the outcome of infants without sepsis.

## Methods

### Research Design and Population

Data from a historical cohort were used. The basic population consisted of all infants born at a gestational age <30 weeks admitted to the NICU of the Academic Medical Center (AMC), Amsterdam, The Netherlands between January 1^st^ 1997 and December 31^st^ 2007.

All neonates from this population with a bacteriological diagnosis of either gram-negative sepsis (GNS) or neonatal Candida sepsis (NCS) (only positive peripheral blood cultures) were included to compare the long- term outcome of these cases.

In order to compare the long- term outcome of GNS and NCS with the outcome of uncomplicated neonatal infants (no sepsis but undergoing identical NICU care) admitted at the same NICU during the same time period and undergoing the same routine care, we randomly selected a set of neonates, without any type of sepsis, from the remaining cases of the same population (UC group). For each case of GNS or NCS, at least 2 control subjects were randomly selected by choosing every other 5^th^ subject from the same basic population. From February to December 2011 clinical and microbiological data were obtained retrospectively from existing written and partly digital medical files, the digital records of the Patient Data Management System (PDMS), the digital clinical chemistry and medical microbiological laboratory databases and the digital long- term follow–up database of the NICU (FUN database).

As existing clinical data were used, collected during the clinical course of these patients and as analyses and reporting of these data was performed anonymously, the approval of the institutional medical ethical committee or signed parental consent were not needed for this research according to Good Clinical Practice - and local legislation, including the Data Protection Act (Wet Bescherming Persoonsgegevens; WBP) and the Medical Contract Bill (Wet Geneeskundige Behandelings-overeenkomst); WGBO.

### Study Population

#### Inclusion and exclusion criteria

Patients were included in the GNS or NCS group *only* when when either gram-negative bacteria or Candida species were found in peripheral blood cultures and if patients were defined as having a clinical sepsis (see below). Only the microorganisms found in the blood cultures were deemed as the causative organism for the sepsis episode.

Patients were *not* included in these study-groups if *only* positive central line cultures were found for either yeast or gram negative bacteria as central line culture results may be misleading.

Clinical sepsis was defined as having at least two of the following symptoms: 1) any increase in frequency or duration of apnoea or bradycardia episodes (not related to feeding problems or airway obstruction) necessitating an increase in ventilatory support (intubation and ventilation; Nasal Intermittent Positive Pressure Ventilation; or increase in Continuous Positive Airway Pressure ); 2) hypothermia (core body temperature of <36.5°C) or hyperthermia (core body temperature of >38.5°C); 3) circulatory compromise: a systolic blood pressure <P5 according to gestational age corrected values.

Patients were included as uncomplicated control group (UC group) subjects if 1) during hospital stay no clinical or bacteriological signs of infection (sterile blood cultures on admission) were identified; 2) either ventilatory support by infant flow CPAP or short (<72 hrs.) ventilation for infant respiratory distress (IRDS) was given and 3) they had received routine NICU care (including indwelling catheters and parenteral nutrition). All infants with possible syndromal traits were excluded.

### Measures

#### Long-term neurodevelopment and outcome measures

Outcome was defined as *adverse* in infants who either 1) died or 2) suffered cerebral palsy either alone or in combination with clinical hearing loss or visual handicaps (mostly ROP) or 3) had MDI/PDI BSID-II-NL results <85.

Post discharge mortality was investigated by evaluating discharge letters and obtaining information from treating physicians in all patients for the follow-up period of the study (24 months corrected age). For all infants the neurodevelopmental outcome was assessed at the corrected age of 24 months by a trained neuropsychologist using the Bayley Scales of Infant Development (Second Edition-Dutch version: BSID-II-NL) [18], [19]. This comprises three separate scales (mental scale, motor scale and behavioral rating). Mental and motor scale scores are converted to a mental developmental index (MDI) and a psychomotor developmental index (PDI) with a mean of 100 and a standard deviation (SD) of 15. Normal limits are defined as MDI and PDI values between 85 and 114. Values of 70–84 are defined as mildly delayed. A score of <70 is defined as severely delayed [20]. The BSID-II has been shown to be reproducible and valid [18], [19]. All infants also underwent a standardised neurological examination performed by trained paediatricians. Some neonatologists of the NICU participate in the long term neonatal follow up program of our hospital. Also digital medical data were available for review for the neuropsychologists and paediatricians performing the outcome assessments. Therefore (and because of the retrospective nature of this study) neurodevelopmental assessment was not blinded for the clinical history.

Outcome was defined as *good* in survivors with a normal neurological examination, no evidence of hearing loss or visual impairment and Mental Developmental Index (MDI)/Psychomotor Developmental Index (PDI) BSID –II-NL score >85. Hearing investigation (newborn hearing screening: digital data) and specialist ophthalmic investigation results (screening for ROP) were digitally available for all cases.

### Clinical Information

Information was retrieved from the medical files on: the time (in days) to the onset of sepsis (counted from the date of admission); total duration of hospitalization (in days), birth weight (grams), gestational age (weeks), Apgar scores, total duration of ventilatory (either intubation and ventilation or CPAP) or inotropic support, total duration of parenteral nutrition and indwelling CVC (all in days). The need for invasive surgical procedures was recorded as well as use of corticosteroid medication.

The following variables were recorded in a dichotome manner: 1) presence of bacterial meningitis; 2) abnormal cerebral ultrasound result (defined as any abnormality detected); 3) presence of retinopathy of prematurity (ROP) [21], [22]; 4) the presence of bronchopulmonary dysplasia (BPD) (defined as the need for supplemental oxygen at 36 weeks postmenstrual age) [23], [24]; 5) abnormal newborn hearing (ALGO) screening result.

To evaluate disease severity at the start of sepsis the Neonatal Therapeutic Intervention Scoring System (NTISS, range 0 to 130) was recorded as a continuous variable. This instrument assesses severity of illness by quantifying the intensity and complexity of care received by a patient. It is based on the assumption that therapeutic intensity is a direct correlate of illness severity, given similar philosophies or styles of care. [25]. The systematic use of the NTISS score was implemented in our unit in 1999.

### Microbiology Data

Microbiological information was recorded on the basis of the blood culture results only. According to the clinical protocol all peripheral blood cultures had been taken prior to start of any antimicrobial therapy. The diagnosis of meningitis was considered as proven only when CSF cultures were positive. Blood cultures were incubated in a semi-automatic system, the BacT/Alert (bioMerieux, Boxtel, the Netherlands). For a blood culture a minimum of 2.0 ml of blood was taken and divided over two bottles (FAN-aerobic: 1.0 ml & FAN-anaerobic: 1.0 ml). Susceptibility to antibacterial and antimycotic agents was determined on Isosensitest and RPMI-plates respectively using disk diffusion (Rosco, Taastrup, Denmark) or on MH plates with E-test (bioMerieux) according to the manufacturers’ instructions. Multiple positive cultures with the same causative microorganism within 7 days were interpreted as one identical sepsis episode. Multiple sepsis episodes were defined as consecutive episodes of clinical sepsis with positive blood cultures with a disease free interval of at least 7 days.

### Statistical Analysis

Statistical analysis was performed with SPSS 18.0 statistical software package (SPSS Inc., Chicago, IL). Independent samples t-tests were used to compare sample means of continuous clinical variables between the GNS and the NCS cohort and between each cohort and the control group. Skewed data were log- transformed. The Mann-Whitney-U test was performed if data were not distributed normally and log transformation did not result in a normal distribution. The Chi squared-test was used for the analysis of nominal data. Differences were considered statistically significant when 2-tailed *p*-values were <0.05.

To analyse the association between the central determinant “sepsis exposure” (GNS, NCS or UC) and outcome (defined as either “adverse” or “good”) multiple logistic regression analysis was performed. Two association models were produced, one crude model unadjusted for confounding and a model adjusted for confounders.

Adjusted estimates for cohort type as a risk factor for adverse outcome were obtained by entering potential confounders in the model one by one. The presence of confounding, defined as a change in the regression coefficient of the central determinant (i.e. cohort type) of >10%, and effect modification, defined as a statistically significant effect of an interaction term, was investigated for all potential confounders in the model in a pre-defined order. Potential confounders were: gestational age; birth weight; total duration of hospitalization (in days); duration of central venous catherization and parenteral nutrition (days); duration of inotropic and ventilatory support (in days); duration of CPAP support (in days); ALGO and ROP screening results; BPD diagnosis at 36 weeks PMA; multiple episodes of infection; neonatal NTISS score and ultrasound result. The presence of co-linearity was investigated by simultaneously adding two co- variables to the regression model that were suspected to cause co-linearity based on a high mutual correlation (e.g. duration of ventilatory and CPAP support). Either a large change in the standard error or a change of the direction of the regression coefficient of these co variables compared with a model containing only one of the 2 co -variables was interpreted as co-linearity.

## Results

### Candida Sepsis, Gram-negative Sepsis and Control Subjects

During the period of 1997–2007, 1362 patients with a gestational age <30 weeks were admitted at the NICU of the AMC. In the study period 57 patients had a positive peripheral blood culture for gram-negative bacteria. Two patients were excluded from this group because there were no clinical signs of sepsis and no positive blood cultures were noted (only a positive central line culture), leaving a group of 55 cases (cumulative incidence 4%). In the same study period there were 31 patients with a peripheral positive blood culture for yeast sepsis; 2 patients were excluded from this group because there were no clinical signs of sepsis and no positive blood cultures were noted (only a positive central line culture), leaving a group of 29 cases (cumulative incidence 2%). There were no patients in our study with only a positive central-line culture and signs of sepsis.

**Figure 1:**
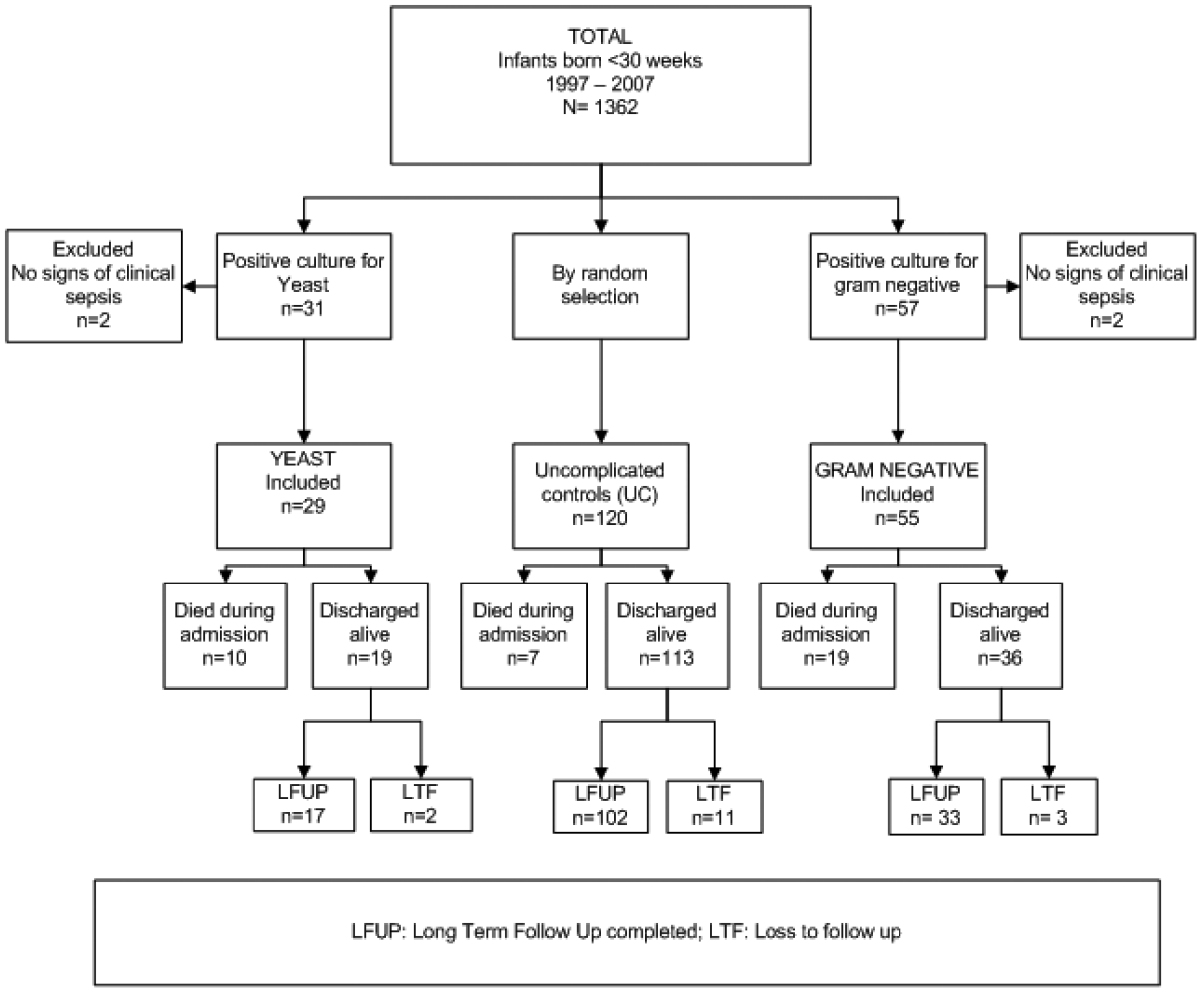
Population Flow sheet. LFUP: Long Term Follow Up completed; LTF: Loss to follow up.

The control group consisted of 120 infants with an uncomplicated clinical course as defined above. The population flow chart is demonstrated in figure 1. Table 1 demonstrates the characteristics of the GNS, NCS and UC groups. Infants who developed GNS and NCS had significantly smaller birth weights than the UC (p<0.001.

**Table 1:** Population Characteristics.

Characteristics	NCS n = 29		GNS n = 55		UC n = 120	
	Mean	SD	Mean	SD	Mean	SD
Birth Weight (grams)	936.6	208.2	933.3	197.8	1130.9	277.6
Gestational Age (weeks)	27.1	1.4	27.0	1.5	28.6	1.2
Apgar score at 10 min	8	2.1	7.9	1.3	8.6	1.1
	**n**	**%**	**n**	**%**	**n**	**%**
Male gender	15	52	34	62	58	48
**Clinical Characteristics**	**Median**	**IQR**	**Median**	**IQR**	**Median**	**IQR**
Days from admission until sepsis episode	16	11–24	17	9–29	n.a	n.a.
Total days of mechanical ventilation	14	6–20	8	3–14	0	0–0
Total days of CPAP	14	6–34.5	26	13–37	5	1–14
Total days of inotropic use	5	0–12.5	1	0–6	n.a.	n.a.
Total days of TPN use	24	13.5–40	20	14–30	8	5–11
Total days of indwelling CVL	27.5	19.25–46.5	24	12–34	7	4.25–10.75
Total days of hospital stay	43	29.5–99.5	50	26.25–69.25	21.5	10–35.75
	**Mean**	**SD**	**Mean**	**SD**		
NTISS	21.7	6.5	23.4	10.2	n.a	n.a
	**n/N**	**%**	**n/N**	**%**	**n/N**	**%**
Postnatal steroids	5/29	17	4/55	7	0/120	0
NEC	6/29	21	10/55	18	1/120	1
CUS normal	13/29	45	34/54^c^	63	91/117^c^	78
CUS abnormal	16/29	55	20/54^c^	37	26/117^c^	22
BPD36	7/29	24	9/55	16	2/120	2
ROP	8/27^c^	30	13/55	24	12/120	10
**Good outcome (normal/mild delay)**	14/27^c^	52%	23/52^c^	44%	92/109^c^	84%
**Adverse outcome (death** * **/severe delay)**	13/27^c^	48%	29/52^c^	56%	17/109^c^	16

c Number of subjects for whom the information was obtained. NCS: Neonatal candida Sepsis; GNS: Gram Negative Sepsis; UC: Uncomplicated Controls; Abbrevations: NEC: surgery for Necrotising Entero Colitis; n.a. = not applicable; IQR = Interquartile Range; SD = standard deviation, BPD36 = diagnosis BPD at 36 weeks, ROP: Retinopathy of Prematurity; NTISS: Neonatal Therapeutic Intervention Score; CUS: cerebral ultrasound; CVL: central venous line; Good outcome = as defined in methods; Adverse outcome = as defined in methods.

* death: all deaths were during admission, no postdischarge mortality.

### Main Study Outcome: Adverse Long-term Neurodevelopment or Demise

Mortality was highly comparable between the GNS and NCS groups (19/55 and 10/29 respectively). All deaths occurred during admission at the NICU.

As Figure 1 demonstrates, the loss to follow–up at the corrected age of 24 months was limited (8%). Table 2 demonstrates the neurodevelopmental outcome results in detail.

**Table 2:** Neurodevelopmental outcome in detail.

Characteristics	NCSn = 29Follow up n: 17	GNSn = 55Follow up n: 33	UCn = 120Follow up n: 102
**Bayley PDI** *	**76 (59–100)**	**84 (52- 110)**	**91 (54- 135)**
**Bayley MDI** *	**92 (78- 108)**	**90 (54- 128)**	**100 (54- 131)**
**Neurology exam^#^**			
Normal	**7/17 (41%)**	**18/33 (55%)**	**87/102 (85%)**
Mildly abnormal	**9/17 (53%)**	**8/33 (24%)**	**13/102 (13%)**
Severely abnormal	**1/17 (6%)**	**7/33 (21%)**	**2/102 (2%)**
**Hearing disabilities**	**4/17 (24%)**	**6/33 (18%)**	**1/102 (1%)**
**Visual handicaps**	**3/17 (18%)**	**10/33 (30%)**	**8/102 (8%)**

NCS: Neonatal Candida Sepsis; Gram Negative Sepsis; UC: Uncomplicated Controls. FU: Follow Up.

* Median, minimum-maximum. ^#^: number of total cases noted and percentage. PDI: Psychomotor Developmental Index; MDI: Mental Developmental Index.

At follow –up of the NCS group normal vision was noted in 82% and normal hearing was observed in 76%. The outcome of the clinical neurological exam was normal in *41%* of the patients.

In the GNS group normal vision was noted in *70%* and in *82%* there was no hearing impairment. The outcome of the clinical neurological examination was normal in *55%* of the patients.

In the uncomplicated control group (UC) 92% had no vision impairment and 99% had no hearing impairment. The outcome of the clinical neurological examination was normal in *85%.*

Combining these findings with the available BSID test results and the mortality results, adverse outcome was noted in 48% of the NCS; 56% of the GNS group and 16% of the UC group.

The statistical analyses demonstrated no statistically significant difference in the odds for adverse outcome (either death or severe neurodevelopmental delay) between the GNS and NCS infants or between the NCS group and the UC group but the OR [95% CI] for adverse outcome in the GNS group compared to the UC group was 4.8 [1.5–15.9]. The crude and adjusted odds ratios (OR) estimated in the regression models are demonstrated in table 3. Effect modification was not found. Co-linearity was found for the duration of indwelling CVC and total duration of parenteral nutrition and for duration of intubation and ventilation and CPAP support. The dichotomous covariable of meningitis and the ordinal covariable “maternal education type” could not be entered in the model as unfortunately >50% of data was missing for both. The events of either surgery for NEC or suffering multiple sepsis episodes did not influence our findings concerning the odds for adverse outcome.

**Table 3:** Raw and adjusted association (Odds Ratios) between sepsis type and adverse outcome.

Cohort type	Crude OR	CI
GNS versus NCS	1.4	0.5–3.5
GNS versus UC	6.8	3.2–14.5
NCS versus UC	5.0	2.0–12.5
	**Adjusted OR**	**CI**
GNS versus NCS	1.4	0.4–4.9
GNS versus UC	4.8	1.5–15.9
NCS versus UC	3.2	0.7–14.7

GNS: gram negative sepsis; NCS: neonatal Candida sepsis; UC: uncomplicated control infants; OR: odds ratio; CI: 95% confidence interval; Adjusted for the following confounders: gestational age; abnormal newborn hearing test; duration of ventilation; total number of days admitted.

### The Clinical Course

Clinical parameters are also shown in table 1. There were no statistically significant differences in any of the clinical parameters between GNS and NCS cases. For gram-negative sepsis *Klebsiella pneumoniae*, *E. coli* and *Enterobacter cloacae* were the most common causative pathogens. The most common causative species for Candida sepsis were *C. albicans* and *C. parapsilosis*. Multiple sepsis episodes occurred in both sepsis groups. In the yeast sepsis group 45% of the patients had experienced multiple episodes of sepsis, compared to 20% of the patients in the gram-negative sepsis group. Table 4 demonstrates the species found in the bloodcultures during these multiple sepsis episodes. During the study period, antimycotic prohylaxis was not prescribed.

**Table 4:** Distribution of Pathogens and Multiple Episodes.

NCS (N = 29)Causative Species	n	%
C. albicans	15/29	(52)
C. parapsilosis	14/29	(48)
**Multiple sepsis episodes; causative m.o.**	13/29	(45)
NCS+Staphylococcal sepsis	3	
NCS+GNS	5	
NCS+GNS+staphylococ sepsis	3	
NCS+GBS sepsis	2	
**GNS (N = 55)** **Causative Species**	**n**	**%**
Klebsiella pneumoniae	13/55	(24)
E. coli	10/55	(18)
Enterobacter cloacae	10/55	(18)
Serratia marcencens	8/55	(15)
Pseudomonas aeruginosa	4/55	(7)
Other	10/55	(18)
**Multiple sepsis episodes; causative m.o.**	11/55	(20)
GNS+Staphylococcal sepsis	10	
Multiple GNS episodes	1	

NCS: Neonatal Candida Sepsis; GNS: Gram Negative Sepsis; GBS: group B streptococcus.

### Retinopathy and Bronchopulmonary Dysplasia

The cumulative incidences of ROP and BPD are shown in table 1. In the GNS group ROP screening results were available for all patients (n = 55). Abnormal results were found in 13 infants (24%) (stage 1: n = 5; stage 2: n = 3; stage 3: n = 5). BPD was diagnosed in 9/55 infants (16%).

In the Candida sepsis group ROP screening results were available for 27/29 infants (93%). Abnormal results were found in 8 infants (30%) (stage 1: n = 3; stage 2: n = 2; stage 3: n = 3). BPD was diagnosed in 7/29 infants (24%).

In the uncomplicated controls ROP screening results were available for all 120 infants. Abnormal results were found in 12 infants (10%) (stage 1: n = 10; stage 2: n = 1; stage3: n = 1). Two infants were diagnosed with BPD (2%).

## Discussion

Our data did not demonstrate any statistically significant difference in the odds for adverse outcome between GNS and NCS cases. In a comparable report by Stoll et al. evaluating outcome of neonatal sepsis in ELBW infants at the corrected ages of 18 and 22 months no significant difference was found between NCS and GNS cases either [3]. Outcome was however not compared to neonatal controls without sepsis in their report. In our study the OR for adverse outcome comparing the GNS and NCS group with UC was high, emphasizing the devastating effect of sepsis on long-term outcome and mortality. It is important to compare our study population to those of previous reports. The incidence of GNS of 4% in our population was comparable to previously reported incidences of 2.7–4.4% [9], [26]. The incidence of NCS in our population was 2%, which is also in accordance with reported incidence of 1.9–9.0% [1], [8], [10]. The mortality rate of 34% for both the GNS and NCS cases was comparable to the mortality ranges as reported previously (19–36%, and 32–44% respectively) [2], [9]–[11]. In line with previous findings, infants suffering clinical sepsis were generally smaller in birth weight than infants with an uncomplicated course [1]–[3], [27].

The distribution of both the Candida species and the gram-negative species in our study population is comparable to previous reports [6], [7], [14]–[16]. This is important in view of the comparison of our results with known international results on outcome of neonatal sepsis cases. During the study period antimycotic prophylaxis was not prescribed to any infant.

The only difference between our study population and earlier described groups seems to be the prevalence of ROP and of BPD. Cumulative ROP incidences have been reported in 51% of children after GNS and in 67–86% of children after NCS [21], [28], [29]. In our study we found a cumulative ROP incidence of 30% in the NCS cohort and of 24% in the GNS cohort, which is lower than previously reported. The high awareness for oxygen radical damage and close ophthalmological monitoring and follow-up in our cohort may have contributed to these low incidences of ROP as continued inflammation and oxygen use are risk factors for sepsis associated morbidities such as BPD and ROP.

Also, the cumulative incidence of BPD in our population, 16% in the GNS cohort and 24% in the NCS cohort, was low compared to previously published reports [3], [30], [31]. This may be explained by the fact that in our study infants below 25 weeks GA with a high risk for pulmonary complications were not present. At that time infants below a gestational age of 25 weeks were not actively treated in the Netherlands.

As far as we know this is the first report comparing outcome between neonates experiencing NCS or GNS with the outcome of uncomplicated controls admitted at the same NICU and treated with the same routine NICU care. This was made possible by highly digitalized databases in which both the clinical course and long- term outcome is registered for the entire NICU population of infants born at a gestational age below 30 weeks. The loss to follow- up in our population was limited as a result of a standardized policy of long term follow- up.

This study has several limitations. Maternal education was not recorded in the early years of the study period and lumbar punctures were not routinely included in the protocol for sepsis work-up until the latter half of the study period. So for both these important factors that may be important for outcome (the presence of meningitis and maternal education) insufficient data were available. This may have influenced our results. Also it is possible that infants with false negative culture results, but with clinical sepsis, were not included in this data set as they were not retrieved by our search from the microbiology databases.

Since this study was performed retrospectively, neurodevelopmental clinicians and neuropsychologists of our outpatient clinic were not blinded for the medical history and outcome evaluation may have differed between sepsis cohort infants and control infants. This information bias may have lead to overestimation of the effect of sepsis.

Another limitation of the study might be that it contains a relatively small number of sepsis cases, especially in the NCS cohort. The comparison of the distribution of clinical characteristics between the NCS cohort and the GNS cohort and the analysis of the long-term outcome demonstrated no statistically significant differences. However, due to the small number of cases a type II error cannot be excluded. From a clinical perspective, the increased ORs for adverse outcome due to a clinical sepsis with inflammatory damage by itself are probably much more important than the specific causative organism as is demonstrated viewing the ORs for adverse outcome comparing both GNS and NCS groups with uncomplicated controls. Clinical experience supports these outcome data. However, larger prospective multicentre studies are needed to construct possible prediction models for neonatal sepsis and its outcome.
